# Functional Outcome of Modified Brostrom-Gould Procedure Using the PopLok Knotless Suture Anchor Technique in Lateral Ankle Instability

**DOI:** 10.7759/cureus.4971

**Published:** 2019-06-22

**Authors:** Mohd Yazid Bajuri, Edewet Daun, Muhammad H Abdul Raof, Mohd Rohaizat Hassan, Srijit Das

**Affiliations:** 1 Orthopaedics and Traumatology, Universiti Kebangsaan Malaysia Medical Centre, Kuala Lumpur, MYS; 2 Community Health, Universiti Kebangsaan Malaysia Medical Centre, Kuala Lumpur, MYS; 3 Anatomy, Universiti Kebangsaan Malaysia Medical Centre, Kuala Lumpur, MYS

**Keywords:** brostrom-gould, suture anchor, ankle, instability, functional, outcome, trauma

## Abstract

Introduction

The modified Brostrom-Gould procedure is surgery to repair the lateral ligamentous complex of an ankle with chronic instability. A retrospective study was carried out among patients who had undergone this procedure at a medical center.

Aim

The aim of the study was to determine the mid-term functional outcome and rate of infection among patients who underwent the surgery using the PopLok® (CONMED, NY, US) Knotless Suture Anchor technique for lateral ankle instability.

Methods

Twenty patients who failed conservative treatment at the Universiti Kebangsaan Malaysia Medical Centre (UKMMC), Kuala Lumpur, and who were operated on by a single surgeon from January 2011 until March 2015 were selected to participate in this study. They were examined clinically both preoperatively and postoperatively and were also evaluated using the American Foot and Ankle Score (AOFAS) and the Visual Analogue Scale (VAS). The last review of the patients, for the purpose of this study, was done at one-year postoperatively.

Results

The patients’ overall AOFAS and VAS scores improved postoperatively as compared to the preoperative period. Preoperatively, the mean AOFAS score was 63.5 while postoperatively, the score was 93.50 (excellent score 90 - 100). The mean VAS score was 8.00 preoperatively and improved to 1.00 postoperatively. There was also an absence of infection observed one-year post-surgery.

Conclusion

The modified Brostrom-Gould procedure using the PopLok® Knotless Suture Anchor technique provides an excellent middle-term functional outcome and a reduction in pain for patients who failed conservative treatment, with a very low rate of infection.

## Introduction

Lateral ankle sprains are one of the most common sports injuries, with approximately 23,000 ankle injuries per day as per reports in the United States [1]. The injury is an inversion type of injury involving the hindfoot in forceful supination on initial ankle dorsiflexion that swings into plantar flexion [2]. In this type of injury, the anterior talofibular ligament (ATFL) is most frequently injured, and in severe sprains, it may be accompanied by injury to the calcaneofibular ligament (CFL) [2].

A surgical procedure using a modified Brostrom-Gould repair is performed to reconstruct the torn lateral ligamentous complex in order to obtain a stable ankle joint. Treating this chronic lateral instability of the ankle joint is important to reduce the risk of osteoarthritis to the ankle joint, to improve the quality of life, and for pain reduction.

This study looked at the functional outcome and postoperative infection rate one year following surgery. Gomez-Carlin et al. [3] studied the functional outcome of 13 patients who underwent a modified Brostrom-Gould procedure and concluded that the surgical procedure showed excellent results in the short term, with the resolution of pain and ankle stability.

This was a pilot study to evaluate the outcome of the modified Brostrom-Gould procedure using the PopLok® Knotless Suture Anchor technique in treating patients with lateral ankle instability due to an ATFL and CFL tear in UKMMC. The main objective of the present study was to determine the functional outcome of the modified Brostrom-Gould repair procedure using the PopLok® Knotless Suture Anchor technique in managing patients with a lateral ligamentous complex injury and chronic symptomatic ankle instability using the American Orthopaedic of Foot and Ankle Society (AOFAS) hindfoot score and the VAS pain score.

## Materials and methods

This was a retrospective study involving patients who underwent the surgical procedure by a single surgeon at a single center (UKMMC) starting from January 2011 to March 2015. Ethical committee approval was obtained from the Research Ethics Committee, The National University of Malaysia (UKM PPI/111/8/JEP-2016-206), prior to the commencement of the study. All patients treated surgically for the lateral ligamentous complex instability of the ankle and treated with modified Brostrom-Gould reconstruction repair after the failure of conservative treatment in which they have completed physiotherapy session and regular pain relief were included. A total of 20 patients were selected.

Data regarding the patients’ demographic features, history, clinical features, and duration of ankle instability were retrieved from the patients’ case notes. Preoperative physical examinations to test for the range of motion and the anterior drawer test [4] assessment were retrieved from the patients’ case notes. A set of accredited questionnaires, which was already filled by the patients prior to the operation, including the American Orthopaedic Foot and Ankle Score (AOFAS) and Visual Analogue Scale (VAS) for pain, was retrieved from the patients' case notes. Informed consent was taken from all patients involved in the study prior to their latest assessment. The same questionnaire was given to the patients on follow-up for comparisons. For the purpose of this study, the final postoperative assessments were all done at one year after surgery. The results of the total AOFAS score were rated as per the protocol: excellent with a score of 90 to 100, good with a score of 70-89, fair with a score of 50 to 69, and poor if the score was less than 50 points. During the one-year post-operative review, the surgical wounds were also inspected for any signs of infection.

All the data were decoded and analyzed using SPSS 22.0 (IBM Corp., Armonk, NY, US), a descriptive analysis was performed using tables and graphs, and an analytical analysis in the form of comparing mean (+SD) or median (interquartile range, IQR) depending on the normality of the data.

**Figure 1:**
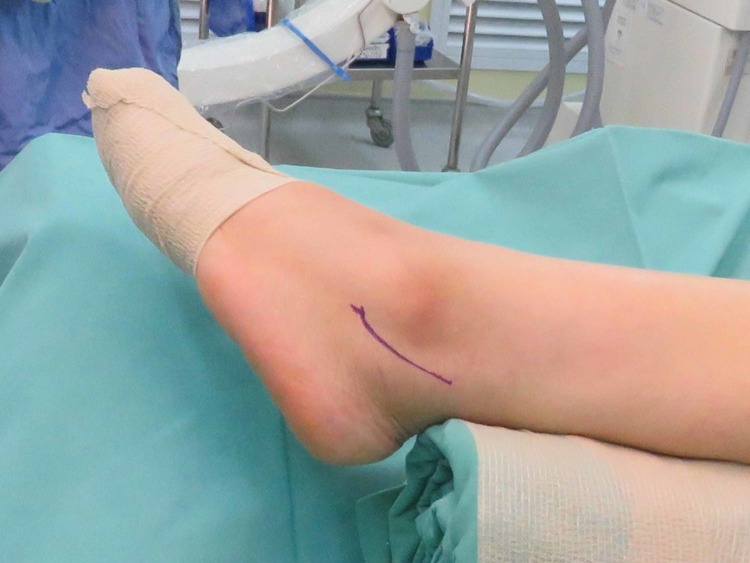
Curve incision made over the inframalleolar region.

**Figure 2:**
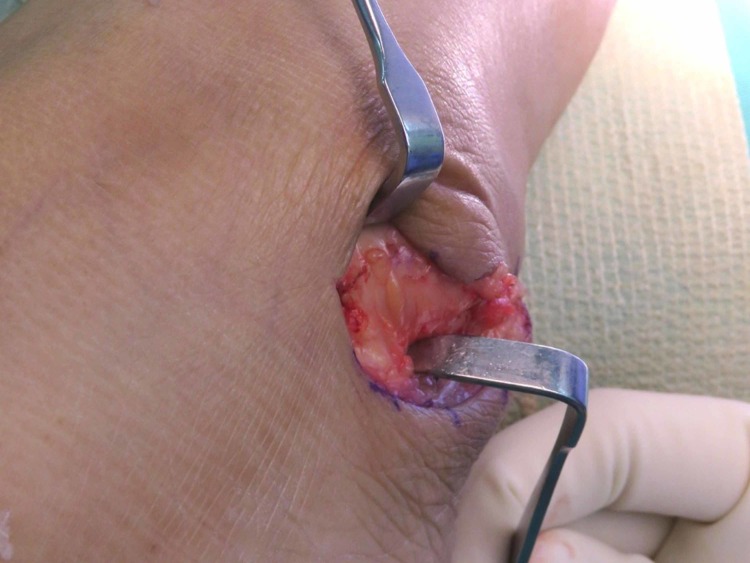
The exposed retinaculum.

**Figure 3:**
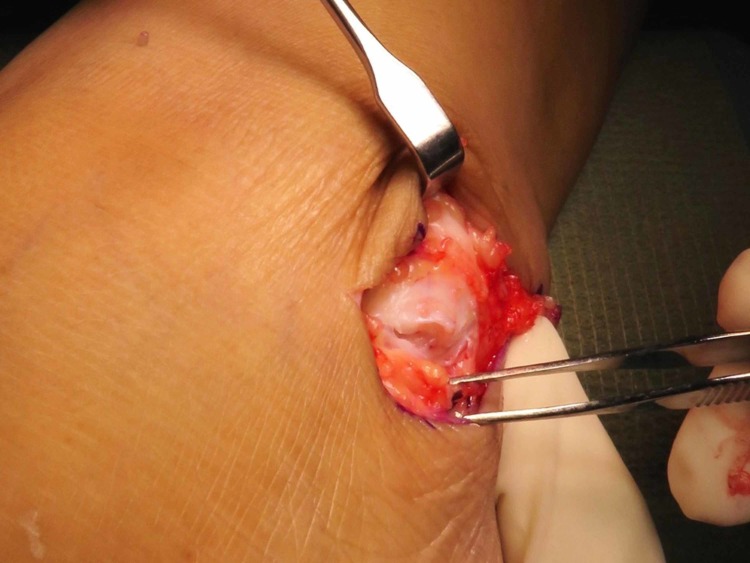
The retinaculum was cut to expose the reconstruction site.

**Figure 4:**
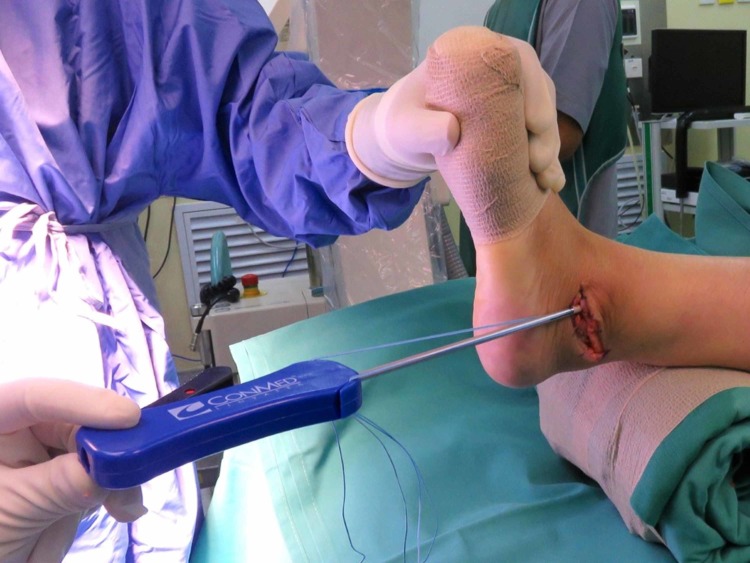
The implant position was marked and checked under image intensifier guidance.

**Figure 5:**
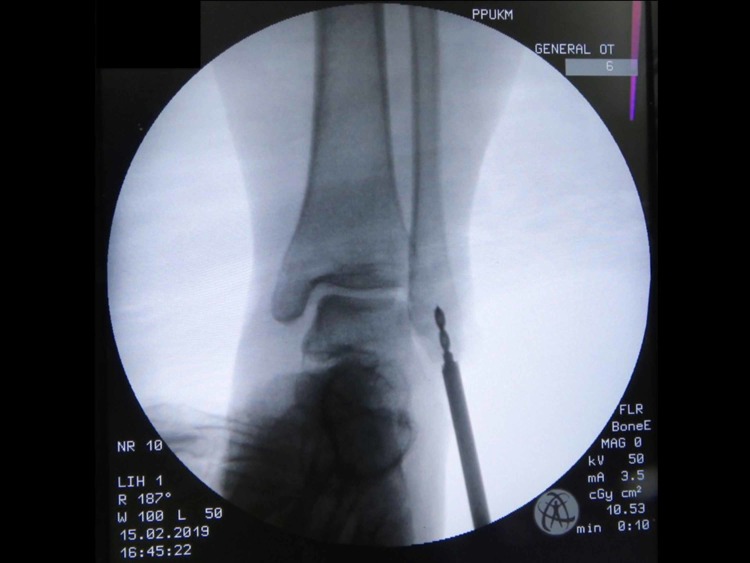
Implant position captured using image intensifier.

**Figure 6:**
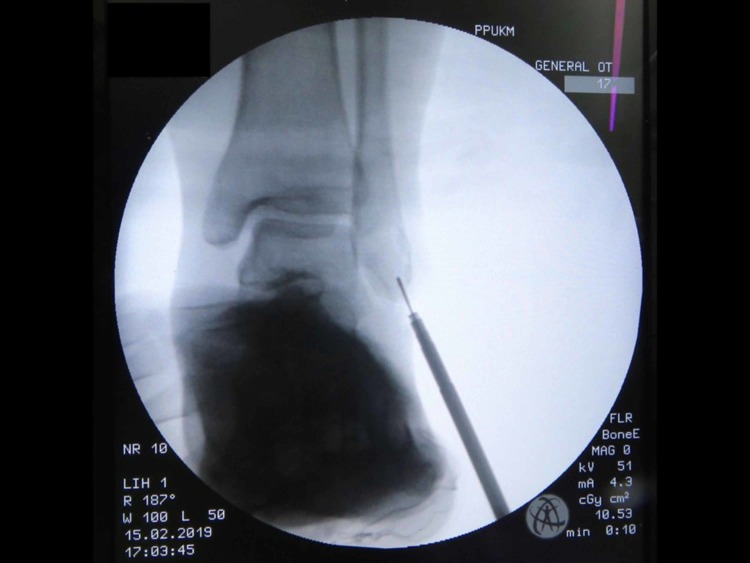
Implant position captured using image intensifier.

**Figure 7:**
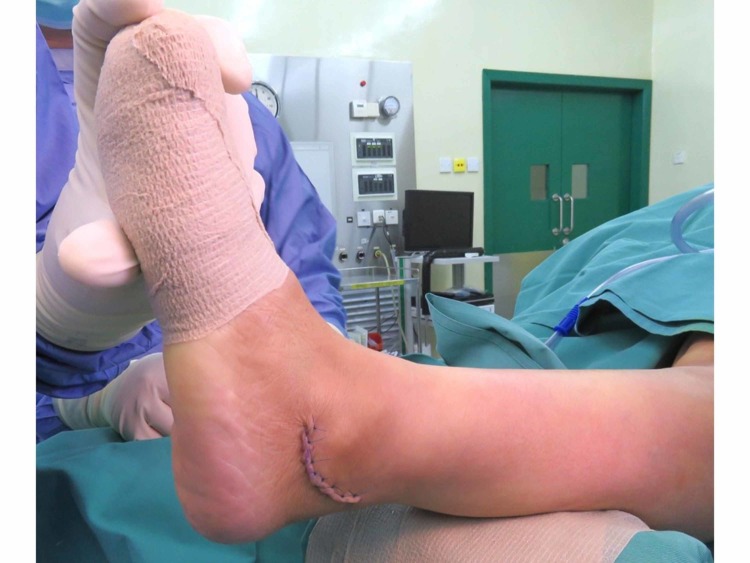
The ankle after closure of the wound; the position of the ankle was maintained in a 10° eversion.

One day prior to the operation, the area of the operative site was washed with chlorhexidine. During the operation, the patient was put in a supine position with her left foot elevated with support using a clean drape (Figure 1) to facilitate the procedure and treatment. A curve incision was made over the inframalleolar region to expose the retinaculum (Figure 2). The retinaculum was then cut to view the lateral ligament complex, mainly the CFL (Figure 3). The CFL was, however, unable to be visualized due to the complete tear, hence a reconstruction was done using the PopLok® Knotless Suture Anchor technique (Figure 4). The position of the PopLok® Knotless Suture was checked with an image intensifier (Figures 5-6). The retinaculum was repaired using Vicryl 2.0 and the wound closure was done in layers (Figure 7).

## Results

A total of 20 patients were included, 13 males and seven females, with a mean age of 37.72 (21-54-year-olds). Eight patients had surgery done on their right ankle and 12 patients on their left ankle. The preoperative mean AOFAS scores (Table 1) was 63.00 (minimum of five, maximum of 71) and the preoperative mean VAS score (Table 2) was 8.00 (minimum of seven, maximum of 10).

**Table 1:** Total AOFAS score pre and post-surgery *Wilcoxon Signed Rank Test; AOFAS: American Foot and Ankle Score; IQR: Interquartile Range

	AOFAS Score		z-value	p-value*
	Mean	IQR		
Pre-Surgery	63.00	52.25-66.50	171.00	<0.001
Post-Surgery	93.50	90.00-94.50		

**Table 2:** Total VAS pain score pre and post-surgery *Wilcoxon Signed Rank Test; VAS: Visual Analogue Scale; IQR: Interquartile Range

	VAS Score		z-value	p-value*
	Mean	IQR		
Pre-Surgery	8.00	7.75-8.00	-3.776	<0.001
Post-Surgery	1.00	0.00-1.00		

Postoperatively, the mean AOFAS score (Table 1) improved significantly to 93.50 (+5.327) while the mean postoperative VAS score (Table 2) was 1.00. In terms of infection, during the last review done at the one-year postoperative period, none was shown to exhibit any signs or symptoms of surgical site infection. Another feedback from the questionnaire was the absence of a suture stump and no feeling of 'grittiness' at the wound area, which increase the patients' satisfaction.

## Discussion

The results of this study showed unequivocally that in cases of lateral ankle instability, the outcome of treatment using the modified Brostrom-Gould procedure using the PopLok® Knotless Suture Anchor technique yielded excellent results. There was a significant increase in the AOFAS score and a decrease in the VAS score at one-year post-surgery. The absence of any infection after one year also indicated the relative safety of the procedure.

The Broström-Gould procedure is a commonly recommended operative treatment for chronic ankle instability [5-9]. Using standardized physician-based outcome scores, the results of this procedure were uniformly excellent. Current scoring systems, however, do not adequately evaluate mechanical or functional instability. Therefore, outcome data may suggest greater success than is justified. A retrospective review was done by Brodsky et al. [10] in 73 patients who had isolated Broström-Gould repairs of the lateral ankle ligaments. The mean time to follow-up was 64 months. Both the AOFAS ankle-hindfoot score and the Short Form 36 (SF-36) were used to evaluate the outcome. The data suggested that greater attention must be paid to functional rehabilitation after ankle stabilization surgery to obtain the optimal outcome. Physical exercise like exercise on a training bike can be added to supplement the postoperative plans as it was observed to have great improvement on the mobility of the ankle joint by a 36.6% increment within nine weeks following routine exercise [11]. This was because the exercise on a training bike helped enhance the functional outcome of the foot postoperatively, as it forced an alternate bending and straightening of the foot. In our study, we also placed emphasis on functional rehabilitation, in which the patient was immobilized for only about four to six weeks depending on the wound healing and then was started with regular physiotherapy sessions to enhance the functional outcome.

In this study, a total of 20 patients were identified: 13 males and 7 female patients. It showed that there was a similar outcome in both gender groups. In a similar study done by Xu et al. [12] to determine whether gender has a significant influence on the outcome of the modified Brostrom-Gould procedure in treating lateral ankle instability, the author came to the conclusion that the modified Broström procedure showed similar good functional and radiographic outcomes in men and women. These results suggest that the modified Broström procedure is effective and reliable for treating chronic lateral ankle instability regardless of gender.

Other studies analyzing Broström surgeries showed good functional outcome. In 2010, a prospective study done by Shahrulazua et al. [13] using bioabsorbable anchors in the modified Broström-Gould surgery showed excellent results for 28 out of 30 ankles. In 2009, a series of 31 patients was published after 27.5 months of arthroscopic repair of the lateral ligament and showed a good clinical result with an AOFAS score of 85.3 [14]. Keller [15] followed 44 patients for 2.5 years with 85% excellent and 13% good results. The complications observed in a few cases were slight wound dehiscences, edemas for more than six months, pain with a recurring sprain, and rigidity. Hennrikus [16] compared the Chrisman-Snook operation with the Broström-Gould surgery and both groups showed 80% excellent or good results. However, more complications were found in patients treated by the Chrisman-Snook procedure. A study done by Fujii [17] while comparing the Broström-Gould procedure to Evans tenodesis on six cadavers reported that the latter procedure gave better stability at the expense of mobility, which was better preserved with the former. Nevertheless, one has to keep in mind that none of the single techniques was able to restore completely contact and motion patterns.

With regard to postoperative infection, Li [18], in a case series of 52 patients, reported a failure rate of 6% (re-rupture) and a 6% complication rate (three cases of superficial wound infection) at a mean of 29-months follow-up. In this pilot study, during the one-year follow-up, no infections were noted. This is achievable by considering the patient’s preoperative condition, as suggested by Bajuri et al. [19], which includes the patient’s underlying metabolic disease and nutritional status prior to the commencement of surgery to reduce the likelihood of infection. However, there is currently no common evaluation score/system available to assess the outcome, hence the presence of disparities in the outcomes of different studies employing different methods of measuring the outcome.

In summary, this pilot study showed that the functional and clinical outcome of the modified Brostrom-Gould procedure using the PopLok® Knotless Suture Anchor technique yielded excellent medium-term results. A limitation of this study, among others, includes that no radiographic comparisons (stress radiographs) were made pre and postoperatively. This was because of the fact that some of the preoperative X-rays were not able to be retrieved from the system for the purposes of the study. The relatively small number of patients (n=20) included in this study is also another limitation. A study with a larger number of patients is necessary for a more accurate assessment. Additionally, in the absence of a universally accepted scoring system, the pre and postoperative clinical assessments might influence the outcome of this study.

## Conclusions

The modified Brostrom-Gould procedure using the PopLok® Knotless Suture Anchor technique yielded excellent medium-term outcomes for patients who had failed conservative treatment. This was shown by significant improvements in both the AOFAS and VAS scores. The absence of infection after one year was also very encouraging. The absence of a suture stump and the associated feeling of ‘grittiness’ secondary to the use of conventional suture techniques increased the patient’s satisfaction postoperatively based on the feedback of the patient.
